# System of Diseases of the Eye

**Published:** 1900-03

**Authors:** 


### Book Reviews.

System of Diseases of the Eye. By American, British, Dutch, French, German and Spanish Authors. Edited by William F. Norris, A. M., M. D., and Charles A. Oliver, A. M., M. D., of Philadelphia, Pa., U. S. A. Volume IV. Motor Apparatus, Cornea, Lens, Refraction, Medical Ophthalmology. With fifty-one full-page plates and 211 text illustrations. 8vo, pp 950. Philadelphia and London: J. B. Lippincott Company. 1900.

The concluding volume of Norris and Oliver's "System of Diseases of the Eye," Vol. IV., is now issued to the medical profession and signals the completion of a monumental work. There has been some delay in its execution and the original arrangement and assignment of articles have been somewhat changed, but in its completed form every department of practical ophthalmology has been adequately treated, and each, for the most part, by a master of the subject in hand.

This volume has sixteen articles, instead of nine, as originally announced. The opening one is on Anomalies of the motor apparatus of the eye, by Edmund Landolt, of Paris, France, translated by C. W. Culver, of Albany, N.Y. This is a subject to which Dr. Landolt has given study and experimental work for years, and he has here, in space of 166 pages, crystalised his conclusions with such clearness and emphasis that, in reading the article, one feels himself in the presence of an authority, such as Dr. Landolt truly is. The first part of the article is devoted to paralysis of the motor muscles of the eye, and deals with its pathology, symptoms (including strabometry), clinical diagnosis, prognosis and treatment, both medical and surgical. The next part treats of nonparalytic strabismus, and enters fully into a consideration of all its varieties, their causation, symptoms, diagnosis and treatment, the latter including the use of spectacles, stereoscopic exercises and operative methods. Incidentally, an excellent review is given of the influence of the so-called "check" ligaments of the eye on the results of tenotomies and advancements. The article concludes with a section on nystagmus, as caused both by disease and occupation. The article is exceedingly interesting and meritorious.

The second article is on the diseases of the cornea, by J. P. Nuel, professor of ophthalmology in the University of Liège, Belgium, translated by Thomas H. Fenton, of Philadelphia, Pa. As the author truly says, "diseases of the cornea constitute one of the most important chapters of ophthalmology, this being due to their serious consequences in regard to sight and their frequency." In this article he has risen to the importance of the subject and has given us, in 86 pages, a complete survey of the diseases of the cornea, their pathology, symptoms, diagnosis and treatment. There are interspersed many beautiful engravings, which throw much light on their pathology. After a short introduction, the author deals with inflammation of the cornea, its general pathology, clinical examination, symptoms of keratitis and general therapeutics. Then he takes up the varieties of superficial keratitis, (*a*) superficial traumatic, (*b*) phlyctenular keratitis, (*c*) granular pannus, (*d*) acne of cornea, corneal herpes, and the like, (*e*) filamentary, (*f*) superficial punctate, (*g*) vesicular bullous and calcareous keratitis, their symptoms, pathology and treatment. Next are the forms of deep keratitis, which the author classified as (*a*) deep ulcerative and suppurative, and (*b*) deep nonsuppurative keratitis, the different forms of which, their symptoms, diagnosis and treatment, the author describes at length. The article concludes with sections on the sequelæ or consequences of keratitis, including cicatricial maculæ of the cornea, keratalgia, sclerosis of the cornea, arcus senilis, corneal staphyloma, corneal fistula, malformations of the cornea, neoplasms and tumors of the cornea and corneal leprosy. This article also has great merit and deserves careful study.

The third article occupies 144 pages and is on Diseases of the lens, by William F. Norris, professor of ophthalmology in the University of Pennsylvania, of Philadelphia, Pa. It is an elaborate chapter on the crystaline lens and its diseases, is beautifully illustrated and is written in a clear and attractive style. The author covers the whole range of affections of the lens, including congenital, traumatic and spontaneous dislocations, malformations, aphakia, and cataract, its etiology, symptoms, diagnosis and treatment. The details of surgical procedures, are not as extended, probably, as would otherwise have been the case, had not these been entrusted to other hands in earlier volumes. But lacking these, the article does not fail to be of great importance, and in view of the author's judicial mind and great experience, his discussion on the comparative merits of the various methods of cataract extraction and on the complications attending operations and their management is invaluable. An excellent section is also included on the removal of the transparent lens for the cure of myopia.

In the next 80 pages, ametropia: its etiology, course and treatment is dealt with in a most clear and precise manner by Charles A. Oliver, of Philadelphia, Pa. The author is an expert refractionist, knows full well the effects of refractive errors upon the human economy, and in this chapter gives the reader the conclusions derived from his wide observation. They are in every way valid and practical.

The remainder of the work may be regarded as a treatise on medical ophthalmology. There are twelve articles as follows: Ocular lesions dependent upon diseases in the circulatory system, by O. Haab, professor of ophthalmology in the University of Zurich, Switzerland, translated by William Zantmayer, of Philadelphia. (58 pages); eye-diseases and eye symptoms in their relation to organic diseases of the brain and spinal cord, by Henry R. Swanzy, surgeon to the Royal Victoria Eye and Ear Hospital, Dublin, Ireland (106 pages); ocular lesions dependent upon disorders of the secretory and excretory organs, by J. B. Lawford, surgeon to the Royal London Ophthalmic Hospital, London, Eng. (42 pages); ocular lesions in variola, rubeola morbilli, scarlatina, erysipelas and diphtheritic, by John B. Story, surgeon to the Royal Victoria Eye and Ear Hospital, Dublin, Ireland (18 pages); ocular lesions of influenza, dysentery, cholera, malarial fever, dengue and yellow fever, by J. Santos-Fernandez, Havana, Cuba, translated by Daniel M. Guitèras (22 pages); ocular manifestations of hysteria, by M. Parinaud, Paris, France, translated by Casey A. Wood, Chicago, Ill. (44 pages); eye-affections, due to Grave's disease and herpes zoster, by Jonathan Hutchinson, Jr., surgeon to the London Hospital, London, England (10 pages); motor changes in the ocular apparatus associated with functional neuroses, by Myles Standish, assistant to the chair of ophthalmology in Harvard University, Boston, Mass. (16 pages); toxic amblyopias, by George E. de Schweinitz, professor of ophthalmology in the Jefferson Medical College, Philadephia, Pa. (46 pages); the entozoa of the human eye, by Maximilian Salzmann, docent for ophthalmology at the University of Vienna, Austria, translated by H. V. Wurdemann, Milwaukee, Wis. (18 pages); simulated blindness, by S. Baudry, professor in the faculty of medicine in the University of Lille, France, translated by Frank W. Marlow, of Syracuse, N. Y. (47 pages); the ocular signs of death, by A. Gayet, professor of clinical ophthalmology to the faculty of medicine in the University of Lyons, France, translated by Edward C. Ellett, of Memphis, Tenn. (14 pages.)

This list of subjects and the eminence of the authors must suffice to indicate to the reader the ground covered and the high character of the contributions made. Not only does the specialist find, here, material of great importance to him, but it is of even greater value to the general practitioner and neurologist.

Taking the "system" as a whole, it seems to us scarcely possible to say too much in its praise. It might not be perfect—the arrangement of subjects might be improved the articles may be proportioned a little more evenly in accordance with their scientific and practical value; a general index to the whole work would increase its convenience for reference, but even with its minor defects, it has no equal in ophthalmological literature in the English language. As we have said before, the progressive ophthalmologist cannot afford to be without it, and it will prove an invaluable addition to every physician's library.

